# Syndrome Keratitis-Ichtyosis-Deafness (KID) chez un enfant togolais issu d'un mariage consanguin

**DOI:** 10.11604/pamj.2015.21.266.6857

**Published:** 2015-08-07

**Authors:** Koussak Kombaté, Bayaki Saka, Dadja Essoya Landoh, Abass Mouhari-Toure, Séfako Akakpo, Eric Belei, Wanguena Gnassingbé, Mohaman Awalou Djibril, Kissem Tchangaï-Walla, Palokinam Pitché

**Affiliations:** 1 Service de Dermatologie-Vénéréologie, CHU Lomé, Faculté des Sciences de la Santé, Université de Lomé, Lomé, Togo; 2 Ministère de la Santé, Division de l'Epidémiologie, Lomé, Togo; 3 Service de Médecine Interne, CHU Lomé, Faculté des Sciences de la Santé, Université de Lomé, Lomé, Togo

**Keywords:** Keratitis-Ichtyosis-Deafness (KID), consanguinité, Lomé Togo, Keratitis-Ichtyosis-Deafness (KID), consanguinity, Lome Togo

## Abstract

Le syndrome KID est une affection génétique rare associant kératite, ichtyose et surdité. Nous rapportons un cas dont la surdité s'est compliquée de mutisme chez un enfant togolais issu d'un mariage consanguin.Il s'agissait d'une fillette de 9 ans admise en dermatologie pour une peau sèche et une kératodermie palmoplantaire évoluant depuis l'enfance, une surdité sévère et un mutisme total évoluant depuis la naissance. Il n'y avait pas d'histoire familiale connue de syndrome KID. Les parents de cet enfant sont des cousins germains. A l'examen, on notait une kératodermie palmoplantaire typique en cuir grossier, une peau sèche ichtyosiforme finement squameuse avec un aspect pachydermique aux genoux et un aspect arlequin aux jambes. L'examen ophtalmologique avait noté une blépharo-conjonctivite, une xérophtalmie, une photophobie et une absence de sourcils. L'examen ORL avait objectivé une hypotrophie des pavillons des oreilles, une surdité sévère et un mutisme total. La particularité de cette observation réside dans la sévérité de l'atteinte auditive qui s'est compliquée de mutisme. Notre enfant étant née de parents consanguins sains, sans histoire familiale de KID, nous pensons que le mode de transmission est probablement sporadique. Une étude moléculaire du cas index et de ses parents, non réalisée à cause de notre plateau technique limité aurait pu le confirmer.

## Introduction

Le syndrome Keratitis-Ichtyosis-Deafness (KID) est une dysplasie ectodermique rare liée à des mutations autosomiques dominantes du gène GJB2 codant pour la connexine 26 [1, 2]. Cliniquement, ce syndrome associe une ichtyose, une kératite et une surdité de perception. C'est une affection sévère dont le retentissement sur la vie sociale est en général majeur. Nous rapportons un cas de syndrome KID associant une atteinte cutanée, oculaire et auditive sévère compliquée de mutisme chez un enfant togolais issu d'un mariage consanguin.

## Patient et observation

**Figure 1:**
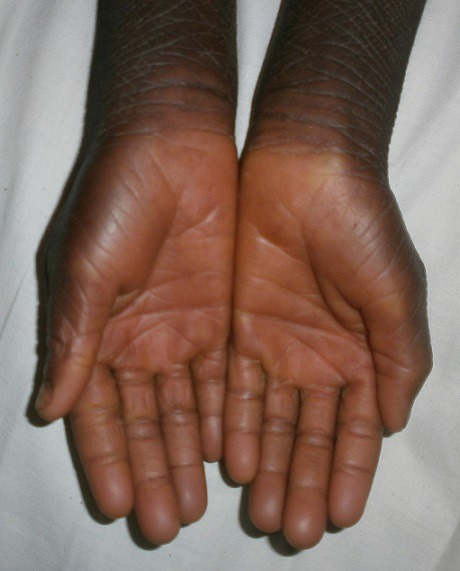
Kératodermie palmaire avec un aspect en cuir grossier

**Figure 2:**
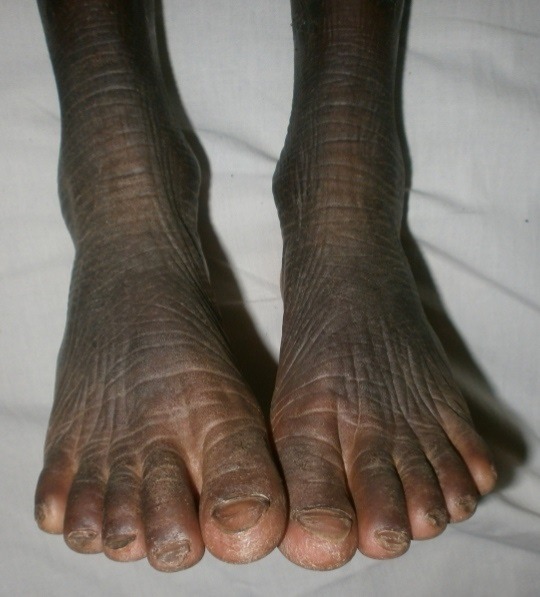
Kératodermie plantaire avec un aspect pachydermique

**Figure 3:**
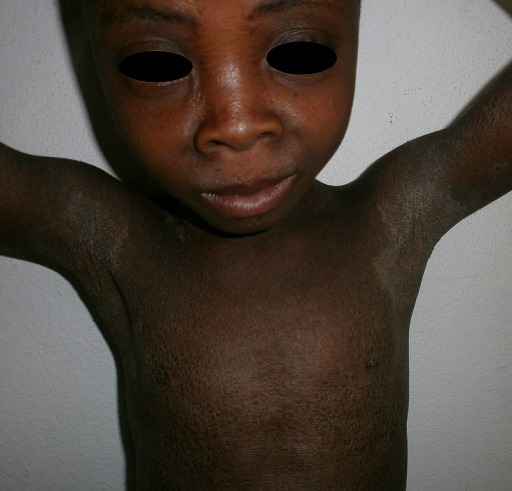
Peau sèche ichtyosiforme finement squameuse; aspect vieillot du visage

**Figure 4:**
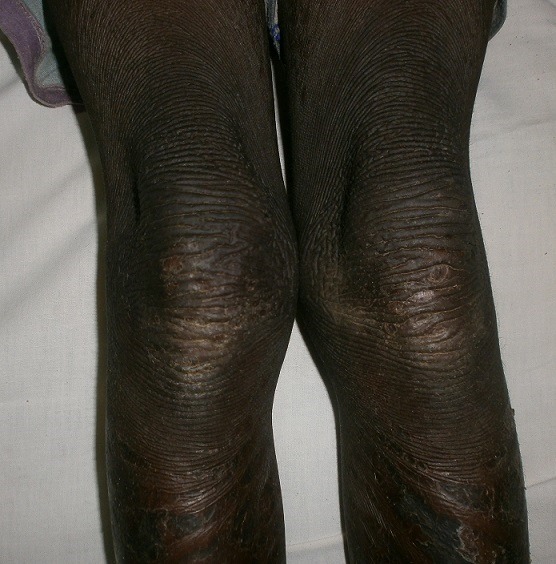
Aspect pachydermique aux genoux et arlequin aux jambes

**Figure 5:**
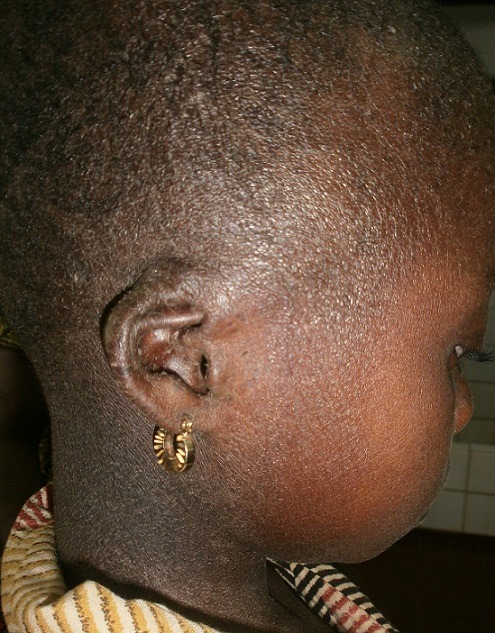
Alopécie diffuse et hypotrophie des pavillons des oreilles

Il s'agissait d'une fillette de 09 ans, admise en dermatologie pour une peau sèche et une kératodermie palmoplantaire évoluant depuis l'enfance, une surdité bilatérale sévère et un mutisme évoluant depuis la naissance. Il n'y avait pas d'histoire familiale connue de syndrome KID. Les parents de cet enfant étaient des cousins germains (la grand-mère paternelle et le grand père maternel étaient des frères). Elle était la deuxième d'une fratrie de deux enfants. L'ainé de sexe masculin était décédé à l’âge de 3 ans. L'enfant a quatre demi-frères de même mère, nés d'un premier mariage avec le grand frère défunt du père de l'enfant (lévirat). Tous les demi-frères sont vivants et sains. A l'examen, l'enfant présentait un retard staturo-pondéral harmonieux avec un bon état général. On notait une kératodermie palmaire (Figure 1) et plantaire typique avec un aspect en cuir grossier Figure 2), une peau sèche ichtyosiforme finement squameuse (Figure 3), avec un aspect pachydermique aux genoux et un aspect arlequin aux jambes (Figure 4). Il y avait également une alopécie diffuse du cuir chevelu, des lésions verruqueuses du visage donnant un aspect vieilli. L'examen ophtalmologique avait noté une blépharo-conjonctivite, une xérophtalmie, une photophobie et une absence de sourcils. L'examen ORL avait objectivé une hypotrophie des pavillons des oreilles (Figure 5), une hypoacousie sévère bilatérale avec une réaction pour un seuil de fréquence correspondant à 80db sur 0,5khz. L'audiométrie tonale n'a pas pu être réalisée à cause du mutisme total. Le reste de l'examen clinique était normal. L'histologie et l'analyse génétique n'avaient pas été réalisées. La patiente était mise sous émollients kératolytiques (Dexéryl^®^), et suivie par un ophtalmologue pour son atteinte oculaire.

## Discussion

Nous rapportons un cas de syndrome KID associant une surdité profonde compliquée de mutisme, une ichtyose sévère avec kératodermie palmoplantaire et une atteinte oculaire chez un enfant né d'un mariage consanguin. L'analyse génétique du cas index et de ses parents qui aurait apporté un argument diagnostic de certitude et confirmer le mode de transmission n'a pas été réalisée à cause de notre plateau technique limité. Le syndrome KID est une affection rare dont la prévalence est difficile à estimer. Dans une revue de la littérature, Cáceres-Rios et al. [3] avaient recensé 61 cas de syndrome KID en 78 ans, de 1915 à 1993. A notre connaissance, cette observation est la seconde au Togo après celle de 2011 [4], mais la quatrième en Afrique Sub-saharienne après les deux cas rapportés au Cameroun en 2013 [5]. Cette affection est le résultat de mutations faux-sens du gène GJB2 codant la connexine 26 [1, 2]. A ce jour, cinq mutations hétérozygotes du gène GJB2 ont été rapportées. La mutation p.Asp50Asn est observée chez environ 81% des patients. Les autres mutations ont été rapportées isolement : p.Asp50Tyr chez un patient [6], p.Gly12Arg chez un patient [1], p.Gly45Glu chez deux patients [7, 8] et p.Ser17Phe chez trois patients [1]. Le mode de transmission du syndrome KID est resté longtemps incertain du fait du faible taux de procréation des sujets atteints en raison de leur aspect très inesthétique. C'est une affection autosomique dominante, mais des possibilités d'un mosaïcisme germinal chez un des deux parents ou d'une pénétrance incomplète ont été évoquées devant la constatation d'une famille comportant deux frères et sœurs atteints issus de parents sains [9]. Chez notre patiente issue de parents consanguins sans contexte familial similaire, le KID syndrome serait probablement sporadique et seule l'analyse génétique du patient et de ses parents aurait pu le confirmer. Sur le plan clinique, contrairement à l'atteinte cutanée, les atteintes oculaire et auditive sont inconstantes au cours du syndrome KID et apparaissent plus tardivement [3, 10]. L'atteinte oculaire, de sévérité variable est caractérisée par une kératite avec des néo-vaisseaux responsable de la cécité. L'atteinte auditive est marquée par une surdité de perception bilatérale [11, 12]. Dans notre observation, la surdité est profonde et compliquée de mutisme puisqu'aucun apprentissage n'a été entrepris préalablement. La prise en charge du syndrome KID est surtout symptomatique et multidisciplinaire. Le traitement de l'atteinte cutanée repose sur les kératolytiques à base d'acide salicylique à 2 %, d'urée à 20 % ou de savons doux. Le traitement des lésions oculaires n'est pas codifié et celui des surdités neurosensorielles est décevant. Enfin, une surveillance régulière s'avère nécessaire pour dépister des carcinomes muqueux.

## Conclusion

Le syndrome KID est une affection génétique rare au carrefour de plusieurs spécialités dont la prise en charge est purement symptomatique. En l'absence d'histoire familiale de syndrome KID, cette observation évoque des possibilités d'un mosaïcisme germinal chez un des deux parents ou d'une pénétrance incomplète. Elle pose le problème des nombreuses affections qui accompagnent les mariages consanguins et incite à les éviter. Il faudra également dépister plus tôt les surdités des nourrissons et les prendre en charge.

**Considération éthique** : nous avons obtenu le consentement éclairé écrit des parents du patient pour la publication de ce cas clinique et les images d'illustration qui accompagnent ce manuscrit.
